# Cholesterol metabolism dysregulated by key HBV mutations revealed through multi-omics profiling

**DOI:** 10.1016/j.isci.2026.115844

**Published:** 2026-04-22

**Authors:** Yimin Chen, Peixia Lin, Jiaxin Jin, Min Deng, Dahai Wei

**Affiliations:** 1 Department of Infectious Diseases, Affiliated Hospital of Jiaxing University, Jiaxing 314001, China; 2 Institute of Liver Disease, Affiliated Hospital of Jiaxing University, Jiaxing 314001, China

**Keywords:** Health sciences

## Abstract

Chronic hepatitis B virus (HBV) infection shows variable progression linked to specific viral mutations. This multi-omics study analyzed 108 patients with chronic HBV, stratified into wild-type, G1896A, A1762T/G1764A, and A1762T/G1764A + G1896A variant groups. Serum proteomics and metabolomics revealed that patients infected with HBV A1762T/G1764A or the double mutants had significantly higher ALT and AST levels, correlating with disease severity. Compared to wild-type, proteomic analysis identified 59, 54, and 79 differentially expressed proteins in the G1896A, double mutant, and combined mutant groups, respectively. Metabolomics showed 201, 242, and 226 altered metabolites in the same groups. Integrated pathway analysis consistently pinpointed cholesterol metabolism as the most disrupted pathway. From this pathway, six key proteins and metabolites emerged as potential combined biomarkers for distinguishing between these HBV variants, linking viral genotype to host molecular response and clinical outcome.

## Highlights

• Cholesterol metabolism is the central dysregulated pathway in key HBV mutant infections
• Key HBV mutations correlate with more severe clinical and molecular disease profiles
• Multi-omics profiling reveals distinct molecular signatures for specific HBV variants
• A six-molecule cholesterol panel shows high diagnostic value for key HBV mutants

## Graphical abstract

**Figure:**
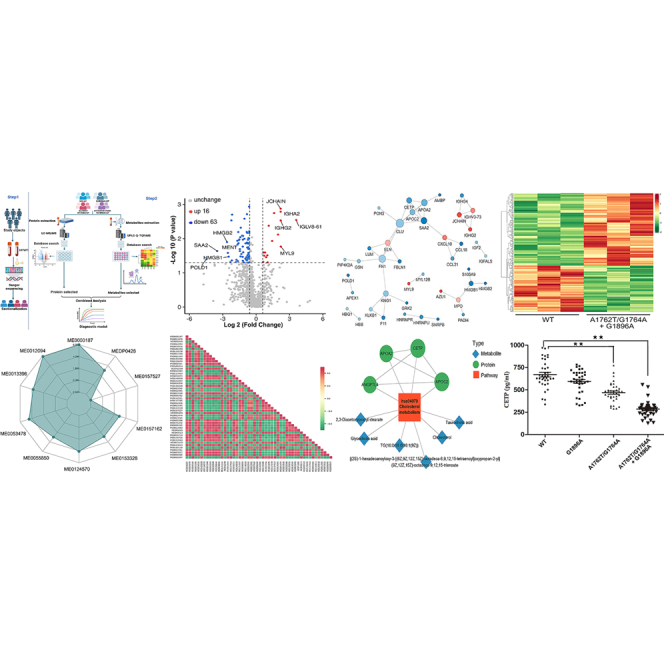

## Introduction

Hepatitis B virus (HBV) infection remains a significant global public health challenge, causing a spectrum of liver diseases ranging from asymptomatic infection and acute hepatitis to chronic hepatitis, which can gradually progress to advanced fibrosis and cirrhosis, and even further trigger hepatocellular cancer (HCC).^1^^,^^2^^,^^3^ According to the World Health Organization statistics, approximately 254 million people worldwide still suffer from chronic HBV infection, with around 1.1 million deaths attributed to HBV-related complications, despite the remarkable reduction in new infections due to widespread vaccination programs and potent antiviral therapies.^4^ The pathogenesis of HBV infection is multifaceted, encompassing both host immune response and viral factors that collectively determine the risk of persistent infection and progression to advanced stages of HBV-related liver diseases.^5^ Specifically, one of the major viral etiologies is the viral mutation, which is correlated with diverse clinical presentations of HBV infection and influences the endemic patterns, transmission dynamics, and liver disease progression and severity.^6^^,^^7^^,^^8^

HBV is an enveloped virus belonging to the Hepadnaviridae family, characterized by a partially double-stranded deoxyribonucleic acid (DNA) genome of approximately 3.2 kilobase pairs containing four overlapping open reading frames (ORFs) denoted as pre-core/core (*PreC/C*) gene, polymerase (*P*) gene, surface gene (*S*), and HBx (*X*) gene, which encode functional viral proteins essential for replication, assembly, and release.^9^^,^^10^ Of note, HBV core antigen (HBcAg) and HBV e antigen (HBeAg) are secreted proteins encoded by the *PreC/C* gene, which are essential viral proteins for the life cycle of HBV and serve as key targets of the host gene regulation and immune recognition to favor HBV persistent infection.^7^^,^^11^ Furthermore, epidemiological observations have also revealed that mutations in the *PreC/C* gene region were the most frequently identified in patients with chronic hepatitis B (CHB), especially after antiviral treatment.^6^^,^^7^^,^^12^ These mutations not only affect the replication and morphogenesis of HBV but may also be associated with the HBV-related liver disease progression by regulating the synthesis of HBcAg and HBeAg and altering the host’s immune response.^6^^,^^7^^,^^8^^,^^13^ Among the myriad mutations in the *PreC/C* gene region, the basal core promoter (BCP) double mutation A1762T/G1764A and the pre-core mutation G1896A are the two most extensively documented and studied HBV mutations related to clinical severity.^6^^,^^7^^,^^12^^,^^13^^,^^14^ As previously stated, A1762T/G1764A mutation reaches a prevalence of 45%–80% in genotypes B and C across East and Southeast Asia as well as sub-Saharan Africa, whereas G1896A mutation dominates genotype D isolates and exhibits a pronounced Mediterranean-to-East Asian gradient.^13^^,^^14^^,^^15^ Functionally, the BCP double mutation A1762T/G1764A reduces HBeAg expression yet simultaneously enhances viral replication via increased pre-genomic RNA transcription, resulting in significantly higher serum HBV DNA levels in HBeAg-negative carriers compared with wild-type (WT) isolates, indicating that the double mutation also abrogates HBeAg-mediated immune tolerance and drives rapid hepatic injury, culminating in acute liver failure and fulminant hepatitis.^11^^,^^12^^,^^13^^,^^14^^,^^15^^,^^16^^,^^17^ As for the PC mutation G1896A, it could cause a premature stop codon (TGG→TAG) at codon 28, thereby abolishing HBeAg synthesis and contributing to the HBeAg-negative phenotype.^11^^,^^12^^,^^13^^,^^14^ In a cross-sectional study completed in Thailand, the A1762T/G1764A double mutation was detected in 44.9% of isolates and independently predicted active CHB (OR = 3.5, 95% CI 1.1–11.3, *p* = 0.037), accompanied by markedly higher HBV DNA (median 4.8 vs. 3.9 log10 IU/mL, *p* = 0.047) and HBV surface antigen (HBsAg) levels (>2,000 S/CO), together with a 5.7-fold increased risk of persistent transaminitis (AST/ALT > ULN) in CHB patients infected with HBV A1762T/G1764A mutants compared to those infected with the WT HBV.^18^ Another study on 322 patients with chronic HBV infection indicated that the prevalence of A1762T/G1764A and G1896A mutations was significantly higher in acute-on-chronic liver failure (ACLF) cases than in CHB patients (50.7% vs. 28.0%, *p* = 0.004; 69.0% vs. 41.5%, *p* = 0.001), with multivariate analysis identifying these mutations as independent risk factors, suggesting that CHB patients carrying these variants have markedly increased susceptibility to ACLF progression compared to those infected with WT strains.^15^ Notably, several studies have also revealed that mutations A1762T/G1764A and G1896A significantly contributed to the progression of chronic HBV infection to HCC and were independently predictive of postoperative survival in HBV-related HCC patients.^6^^,^^11^^,^^16^^,^^19^^,^^20^^,^^21^ A study of 26,178 consecutive pregnant women in China revealed that A1762T/G1764A and G1896A HBV mutations increased in prevalence with age among children aged 1–15 years, suggesting immune selection pressures during early infection, while these HCC-associated HBV mutations were found to be less frequent in children than in their mothers, indicating stage-specific differences in mutation frequencies within the same viral population.^21^ Therefore, these findings strongly suggest that infections with HBV A1762T/G1764A and G1896A mutants might be more aggressive with regard to HBV-related liver disease, compared with WT infections.

After hepatocyte infection, HBV transfers its relaxed circular DNA into the nucleus, where it can integrate into the host genome and exploit the host’s transcription-translation machinery and energy metabolism to complete its replication.^3^^,^^22^ Generally, HBV infection alters host macromolecular and metabolic synthesis of related substances, which can, in turn, modify the synthesis of proteins and metabolites in the host by inducing systematic reprogramming in the host proteomic and metabolic profile and optimizing the conditions for persistent infection.^10^^,^^23^ Therefore, these reprogramming events may induce alterations in host proteomic and metabolic profiles, as well as their regulatory networks, thereby facilitating HBV infection and driving the progression of HBV-related liver diseases. For instance, recent studies have revealed that the A1762T/G1764A mutation selectively upregulates pre-genomic RNA transcription while suppressing *PreC* mRNA expression, thereby enhancing HBV DNA synthesis via reverse transcription and increasing viral replication efficiency by creating novel binding sites for the hepatocyte-specific transcription factors hepatocyte nuclear factor 1α (HNF-1α) and hepatocyte nuclear factor 1β (HNF-1β) in the BCP region, with HNF-1α further promoting HBV replication and enabling immune evasion while concurrently reducing HBeAg and HBsAg levels.^24^ Accumulating evidence demonstrates that the G1896A mutation significantly decreases the expression of HLA class II molecules *in vitro* while activating both endoplasmic reticulum (ER) stress responses and TNF signaling pathways, ultimately triggering more extensive and severe hepatocyte injury, even promoting tumorigenesis and inhibiting apoptosis in hepatoma cells through ERK/MAPK pathway activation.^25^^,^^26^^,^^27^ These findings imply that specific differences in molecular pathogenesis during HBV infection among patients infected with HBV G1896A, A1762T/G1764A, and A1762T/G1764A + G1896A mutants and WT strains indeed exist and remain to be fully elucidated.

High-throughput detection technologies enable comprehensive profiling of protein and metabolite targets by simultaneously quantifying multiple analytes in blood samples, with these methods being widely applied to investigate host-pathogen interactions during viral infections across humans, animals, and plants.^28^^,^^29^^,^^30^^,^^31^^,^^32^^,^^33^ Meanwhile, the integrative proteomic and metabolomic strategy enables simultaneous quantification and comparative analysis of multiclass proteins and metabolites, offering a systems-level framework for forward-looking discovery of diagnostic biomarkers and therapeutic targets in various diseases.^32^ Thus, the clinical proteomic-metabolomics approach offers a comprehensive view of host factors in viral infection, revealing signaling pathway disruptions and advancing us to further understand the molecular pathogenesis of HBV infections. Here, the objective of this study is to elucidate the molecular mechanisms by which HBV G1896A, A1762T/G1764A, and A1762T/G1764A + G1896A mutations modulate viral replication, immune evasion, and liver disease progression in CHB patients. To achieve this, we utilized data-independent acquisition (DIA) in liquid chromatography-tandem mass spectrometry (LC-MS/MS)-based serum proteomic and ultra-high-performance liquid chromatography quadrupole-orbitrap tandem mass spectrometry (UHPLC-Q-Orbitrap MS/MS)-based serum metabolomic techniques to obtain unbiased, differentially expressed protein and metabolic profiling data among patients infected with HBV A1762T/G1764A and G1896A mutants. The findings from this study will elucidate the pathogenic mechanisms and provide foundational insights for targeted therapies and further deep study in CHB patients carrying HBV G1896A, A1762T/G1764A, and A1762T/G1764A + G1896A mutants.

## Results

### Clinical characteristics of the study participants

As described in Figure S1, 252 serum samples were collected, all of which were successfully amplified across the BCP/PC region and yielded high-quality sequencing data with clear chromatograms. Representative sequencing profiles are provided in Figure S2, establishing a robust foundation for subsequent analysis. In each group categorized by HBV mutation status, the patients were subsequently randomized into four subgroups, with nine individuals per subgroup (*n* = 27). Baseline characteristics including age, sex distribution, body mass index (BMI), alkaline phosphatase (ALP), albumin (ALB), international normalized ratio (INR), and fibrinogen (FIB) demonstrated no statistically significant differences among the four groups. However, comparative analysis revealed significant differences in several laboratory parameters between the WT group and the three mutation groups (HBV G1896A, A1762T/G1764A, and A1762T/G1764A + G1896A). Specifically, these included alanine aminotransferase (ALT), aspartate aminotransferase (AST), gamma-glutamyl transferase (GGT), total bilirubin (TBIL), direct bilirubin (DBIL), total bile acid (TBA), prothrombin time (PT), HBsAg levels, HBeAg status, and HBV DNA viral load (Table 1).

**Table 1:** Comprehensive baseline characteristics of patients enrolled in this study

Characteristic	Wild-type	G1896A	A1762T/G1764A	A1762T/G1764AG + 1896A
Age (years)	46.60 ± 13.38	43.01 ± 13.32	42.30 ± 10.14	40.89 ± 11.87
Male, n (%)	15 (55.6)	19 (70.3)	16 (59.2)	19 (70.3)
BMI (kg/m^2^)	24.78 ± 4.60	24.21 ± 4.15	24.38 ± 3.50	23.47 ± 3.11
PLT (×10^9^/L)	145.33 ± 94.59	167.60 ± 83.78	151.07 ± 60.12	160.83 ± 68.17
ALT (IU/L)	162.0 (41.5, 288.0)	198.0 (123.5, 454.0)^a^	272.0 (138.0, 687.5)^b^	309.0 (223.2, 1185.2)^c^
AST (IU/L)	122.0 (61.0, 207.5)	173.0 (100.5, 283.5)^a^	240.0 (148.0, 461.0)^b^	419.5 (116.5, 581.8)^c^
GGT (IU/L)	88.0 (47.5, 245.0)	142.0 (68.5, 240.5)^a^	142.0 (67.0, 262.6)^b^	104.0 (74.5, 166.0)^c^
TBIL (μmol/L)	21.0 (14.4, 33.0)	26.2 (16.9, 30.0)^a^	34.0 (25.8, 42.4)^b^	44.9 (27.4, 127.4)^c^
DBIL (μmol/L)	9.0 (5.9, 18.1)	12.5 (6.2, 23.9)^a^	16.0 (9.4, 30.9)^b^	32.4 (13.9, 102.2)^c^
TBA (μmol/L)	24.0 (10.9, 116.9)	37.4 (8.2, 152.1)^a^	80.2 (47.3, 167.9)^b^	70.1 (21.6, 241.3)^c^
ALP (IU/L)	121.59 ± 82.09	168.10 ± 100.68	142.30 ± 26.45	148.14 ± 42.32
ALB (g/L)	34.65 ± 7.70	39.16 ± 5.81	35.47 ± 6.71	37.54 ± 5.86
PT (s)	14.37 ± 2.85	16.59 ± 3.50∗	17.05 ± 2.07^b^	19.77 ± 2.83^c^
INR	1.1 (1.0, 1.8)	1.1 (1.0,1.4)	1.2 (1.0,1.3)	1.1 (1.0,1.2)
TT (s)	19.56 ± 2.71	19.27 ± 1.62	19.91 ± 1.38	19.81 ± 1.61
APTT (s)	35.96 ± 3.64	39.21 ± 5.01	41.01 ± 5.13^b^	42.41 ± 5.43^c^
FIB (g/L)	2.62 ± 1.62	2.85 ± 1.41	2.32 ± 1.38	2.30 ± 1.59
HBsAg (IU/mL)	829.8 (7.3, 2129.9)	942.6 (405.4, 5942.2)^a^	1013.4 (475.7, 15450.4)^b^	2690.8 (1250.0, 12189.6)^c^
HBeAg (+), n (%)	23 (85.2)	12 (44.4)^a^	10 (37.0)^b^	9 (33.3)^c^
HBcAb (IU/mL)	6.73 ± 1.91	7.24 ± 1.95	7.57 ± 2.65^b^	11.33 ± 6.6^c^
HBV viral DNA (log_10_)	3.82 ± 2.08	4.76 ± 2.13	5.90 ± 2.12^b^	6.77 ± 1.49^c^

BMI, body mass index; PLT, platelets; ALT, alanine aminotransferase; AST, aspartate aminotransferase; GGT, gamma-glutamyl transferase; TBIL, total bilirubin; DBIL, direct bilirubin; TBA, total bile acid; ALP, alkaline phosphatase; AlB, albumin; PT, prothrombin time; INR, international normalized ratio; APTT, activated partial thromboplastin time; FIB, fibrinogen; HBsAg, HBV surface antigen; HBeAg, hepatitis B e antigen; HBcAg, HBV core antigen.

a *p* < 0.05 for comparisons between G1896A and WT.

b *p* < 0.05 for comparisons between A1762T/G1764A and WT.

c *p* < 0.05 for comparisons between A1762T/G1764A + G1896A and WT.

### Serum proteomic profiling of patient infected with HBV A1762T/G1764A and G1896A mutants

**Figure 1:**
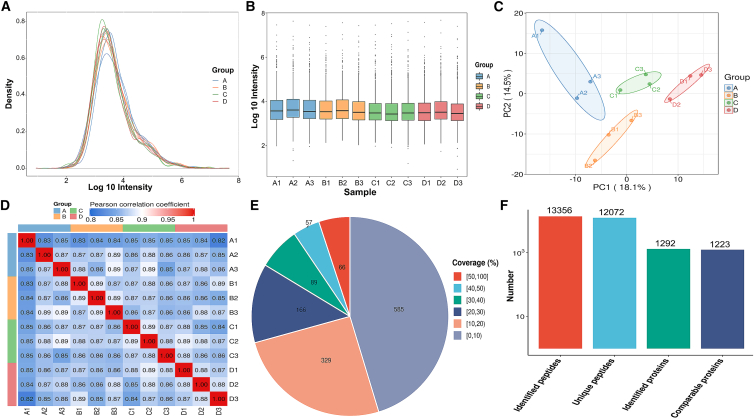
Quantitative proteomic analysis based on DIA in LC-MS/MS (A) Protein abundance distribution curve. (B) Boxplot of protein abundance distribution. (C) Principal-component analysis (PCA) plot. (D) Pearson’s correlation analysis plot. (E) Protein coverage distribution. (F) Overall statistics of identified peptides and proteins.

To characterize the protein profiles of serum samples, we performed a comprehensive quality control (QC) analysis that validated robust analytical performance, as evidenced by: (1) consistent intensity distributions in boxplots (Figure 1A) and density curves (Figure 1B), showing uniform intensity distributions; (2) tight sample clustering in principle-component analysis (PCA) (Figure 1C), reflecting instrument stability; and (3) inter-group Pearson’s correlation coefficient > 0.85 (Figure 1D), demonstrating high experimental reproducibility. In our study, we identified 1,292 unique proteins from 13,356 peptides in the UniProt human proteome database, and after filtering out high-abundance proteins based on protein coverage distribution (Figure 1E), we further performed quantitative analysis on a total of 1,223 proteins (Figure 1F).

**Figure 2:**
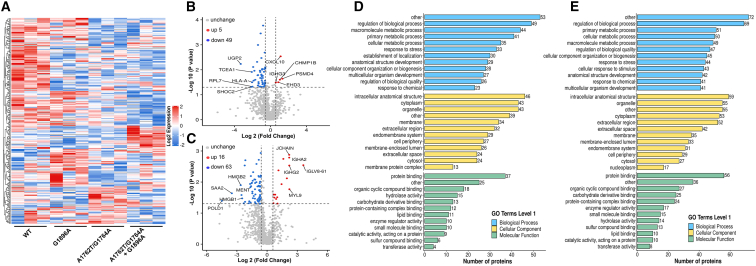
Bioinformatics analysis of differentially expressed proteins in serum from patients infected with HBV A1762T/G1764A (group C) and A1762T/G1764A + G1896A (group D) mutants versus wild-type controls (group A) (A) Hierarchical clustering analysis of dysregulated serum proteins. (B) Volcano plot showing changes in protein abundance (group C vs. group A). (C) Volcano plot showing changes in protein abundance (group D vs. group A). (D) GO analysis of the 54 dysregulated proteins (group C vs. group A). (E) GO analysis of the 79 dysregulated proteins (group D vs. group A).

To identify differentially expressed proteins, we performed pairwise comparisons of relative protein expression values between the mutational groups (G1896A, A1762T/G1764A, and A1762T/G1764A + G1896A) and the control group (WT). Compared with the WT group, 59 differentially expressed proteins (16 upregulated and 43 downregulated) (Figures 2A and S3A; Table S1) were identified in the G1896A group, while 54 proteins (5 upregulated and 49 downregulated) (Figures 2A and 2B; Table S2) showed differential expression in the A1762T/G1764A group, all with a mean fold change ≥1.5 and *p* < 0.05. The list of typically differentially expressed proteins is provided in Table 2. Functional annotation via Gene Ontology (GO) enrichment analysis classified the dysregulated proteins into biological processes (BP) and cellular compartments (CC) categories, revealing their biological significance and subcellular localization. In the BP category, the most significantly enriched terms were regulation of biological process, macromolecule metabolic process, and cellular component organization or biogenesis, whereas the proteins with differential abundance were predominantly associated with MFs, including protein binding, organic cyclic compound binding, hydrolase activity, and carbohydrate derivative binding (Figures 2D and S3B).

**Table 2:** List of typically differentially expressed proteins between the serum samples from patients infected with HBV G1896A, A1762T/G1764A, and A1762T/G1764A + G1896A mutants

Protein accession	Gene name	Protein description	Fold change G1896A vs. WT	Fold change A1762T/G1764A vs. WT	Fold change A1762T/G1764A + G1896A vs. WT
P0DJI9	SAA2	serum amyloid A-2 protein	1.13	0.10	0.09
P26583	HMGB2	high-mobility group protein B2	0.21	0.31	0.16
P06702	S100A9	protein S100-A9	0.14	0.40	0.21
P02760	AMBP	protein AMBP	0.70	0.49	0.22
Q9BXJ9	NAA15	N-alpha-acetyltransferase 15, NatA auxiliary subunit	0.52	0.36	0.33
P11233	RALA	Ras-related protein Ral-A	0.23	0.31	0.33
P03951	F11	coagulation factor XI	0.48	0.37	0.36
P08237	PFKM	ATP-dependent 6-phosphofructokinase	0.33	0.17	0.37
Q8NBZ7	UXS1	UDP-glucuronic acid decarboxylase 1	0.44	0.66	0.37
Q9BY76	ANGPTL4	angiopoietin-related protein 4	0.54	0.50	0.42
P11597	CETP	cholesteryl ester transfer protein	0.89	0.64	0.43
Q9UM07	PADI4	protein-arginine deiminase type-4	0.29	0.54	0.44
P02652	APOA2	apolipoprotein A-II	0.60	0.50	0.52
P02655	APOC2	apolipoprotein C-II OS	0.88	0.64	0.56
P01042	KNG1	kininogen-1	0.76	0.72	0.61
P48426	PIP4K2A	phosphatidylinositol 5-phosphate 4-kinase type-2 alpha	0.82	1.04	0.65
P15502	ELN	elastin	1.01	1.35	1.60
Q9NZN3	EHD3	EH domain-containing protein 3	2.51	1.82	1.65
P55036	PSMD4	26S proteasome non-ATPase regulatory subunit 4	0.85	2.50	1.87
P60981	DSTN	destrin	2.62	2.50	2.71

Furthermore, a comparative analysis of serum protein expression between the A1762T/G1764A + G1896A and WT groups identified 79 dysregulated proteins (16 upregulated and 63 downregulated) per established criteria (Figures 2A and 2C; Table S3). Systematic GO analysis demonstrated three-tiered functional patterns: BP was dominated by the regulation of biological process (69 proteins), primary metabolic process (51 proteins), and cellular metabolic process (50 proteins); CC analysis revealed cellular compartmentalization, with intracellular anatomical structure (59 proteins), organelle (55 proteins), and cytoplasm (53 proteins) constituting the major subcategories; and MF distribution was characterized by protein binding (56 proteins), organic cyclic compound binding (27 proteins), carbohydrate derivative binding (25 proteins), and protein-containing complex binding (24 proteins) (Figure 2E). Collectively, our results revealed that HBV G1896A, A1762T/G1764A, and A1762T/G1764A + G1896A mutations induce mutation-specific regulatory networks in biological processes and molecular functions, suggesting that divergent molecular mechanisms contribute to their distinct pathogenic profiles compared to WT HBV infection.

### Correlation and KEGG pathway analysis of the differentially expressed proteins

**Figure 3:**
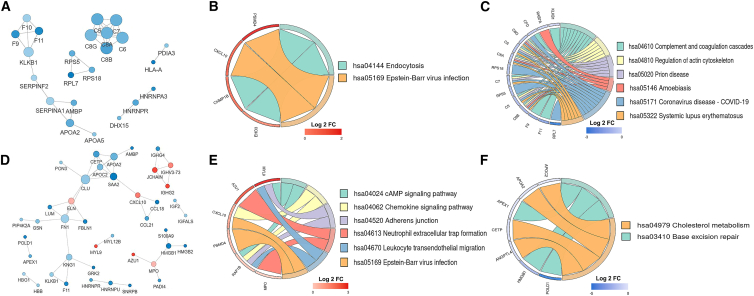
Functional enrichment analysis of differentially expressed proteins in serum from patients infected with HBV A1762T/G1764A and A1762T/G1764A + G1896A mutants (A) Protein-protein interaction (PPI) network of the most closely interacting proteins in HBV A1762T/G1764A mutation group. (B) Chord diagram showing significantly differentially expressed proteins in the HBV A1762T/G1764A mutation group and their corresponding upregulated KEGG pathways. (C) Chord diagram showing significantly differentially expressed proteins in the HBV A1762T/G1764A mutation group and their corresponding downregulated KEGG pathways. (D) PPI network of the most closely interacting proteins in the HBV A1762T/G1764A + G1896A mutation group. (E) Chord diagram showing significantly differentially expressed proteins in the HBV A1762T/G1764A + G1896A mutation group and their corresponding upregulated KEGG pathways. (F) Chord diagram showing significantly differentially proteins in the HBV A1762T/G1764A + G1896A mutation group and their corresponding downregulated KEGG pathways.

To elucidate the functional roles of proteins exhibiting HBV mutation-associated expression changes, we performed Kyoto Encyclopedia of Genes and Genomes (KEGG) pathway enrichment analysis on these dysregulated proteins, which were at the core of the protein-protein interaction (PPI) network (Figures 3A, 3D, and S3C). KEGG pathway enrichment analysis revealed that dysregulated proteins in HBV G1896A group patients’ serum were predominantly associated with Ras signaling pathway, mitophagy, and amoebiasis, whereas those in HBV A1762T/G1764A group patients’ serum primarily participated in endocytosis, complement and coagulation cascades, and regulation of actin cytoskeleton (Figures 3B, 3C, and S3D). In contrast, the dysregulated proteins were predominantly enriched in pathways associated with cAMP signaling pathway, chemokine signaling pathway, and cholesterol metabolism when comparing HBV A1762T/G1764A + G1896A mutation groups with the WT HBV group (Figures 3E and 3F). Thus, the results demonstrated that specific signaling pathways were indeed involved in the molecular differences of host cellular processes between patients in the HBV mutational groups (G1896A, A1762T/G1764A, and A1762T/G1764A + G1896A) and those in the WT group, despite the identification of some common signaling pathways.

### Serum untargeted metabolomic profiling of patients infected with HBV A1762T/G1764A and G1896A mutants

**Figure 4:**
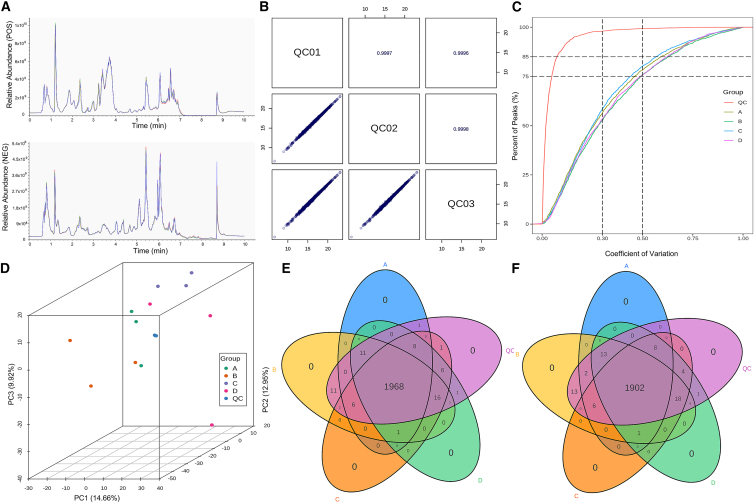
Quality control analysis of serum metabolites from patients infected with HBV A1762T/G1764A and A1762T/G1764A + G1896A mutants (A) Overlaid total ion chromatograms (TICs) of QC samples from mass spectrometry analysis. (B) Pearson’s correlation analysis of QC samples. (C) Distribution of coefficient of variation (CV) values across all samples. (D) Principal-component analysis (PCA) of all samples. (E) Venn diagram showing the number and overlap of metabolites identified in the four groups under positive (POS) ionization modes. (F) Venn diagram showing the number and overlap of metabolites identified in the four groups under negative (NEG) ionization modes.

Untargeted metabolomic analysis of serum samples from four experimental groups was conducted in both positive (POS) and negative (NEG) ionization modes. The total ion chromatograms (TICs) demonstrated excellent signal overlap across the samples (Figure 4A), with a Pearson’s correlation coefficient of 0.997 for QC)samples (Figure 4B), indicating high analytical consistency. Notably, over 75% of detected metabolites exhibited low variability, as reflected by coefficients of variation (CVs) below 0.3 (Figure 4C). PCA further confirmed the tight clustering of QC samples (Figure 4D), underscoring exceptional instrument stability and reproducibility. Untargeted metabolomic analysis with three biological replicates revealed comprehensive metabolic profiles across four experimental groups: 1,995 metabolites were quantified in the WT group, 2,015 in G1896A, 2,008 in A1762T/G1764A, and 2,015 in the A1762T/G1764A + G1896A dual-mutant group. Notably, 1,968 overlapping metabolites (96.71% of total detected compounds) were consistently identified in all groups under POS ionization mode (Figure 4E). Parallel analysis in NEG ionization mode yielded 1,932, 1,964, 1,939, and 1,956 metabolites for respective groups, with 1,932 common metabolites (96.21% coverage) demonstrating robust reproducibility across the samples (Figure 4F). Collectively, these results validated the methodological robustness of the analytical model, fulfilling the acceptability criteria for subsequent differential metabolite identification and intergroup classification, while the observed high reliability and predictive power support its applicability for subsequent metabolomic profiling.

**Figure 5:**
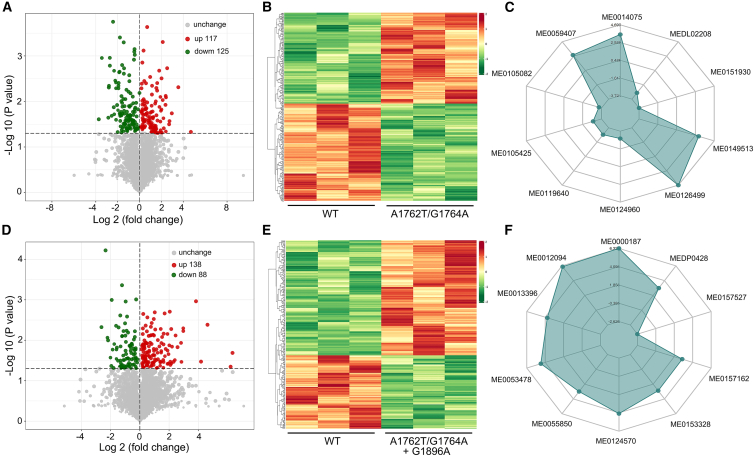
Bioinformatics analysis of differentially expressed metabolites from patients infected with HBV A1762T/G1764A and A1762T/G1764A + G1896A mutants (A–C) Volcano plot showing differences (A), hierarchical clustering (B), and radar chart (C) of top 10 differentially expressed metabolites in serum samples from the HBV A1762T/G1764A mutation group and WT group (group C vs. group A). (D–F) Volcano plot showing differences (D), hierarchical clustering (E), and radar chart (F) of top 10 differentially expressed metabolites in serum samples from the HBV A1762T/G1764A + G1896A mutation group and WT group (group D vs. group A).

To identify differentially expressed serum metabolites, we compared the relative abundance values of metabolites in HBV mutational groups (G1896A, A1762T/G1764A, and A1762T/G1764A + G1896A) against the WT HBV group. Significantly altered metabolites meeting predefined thresholds (variable importance in projection [VIP] value >1 and *p* value < 0.05) were subsequently normalized by unit variance (UV) scaling and analyzed via *K*-means clustering, which revealed distinct, well-separated metabolite clusters, consistent with genotype-specific metabolic patterns as expected (Figure S4). Hierarchical clustering analysis revealed 201 differentially expressed metabolites (111 upregulated and 90 downregulated) in HBV G1896A (Figure S5A and Table S4), 242 (117 upregulated and 125 downregulated) in A1762T/G1764A (Figure 5A and Table S5), and 226 (138 upregulated and 88 downregulated) in A1762T/G1764A + G1896A (Figure 5D and Table S6) groups compared to the WT HBV group. Notably, these metabolite subsets formed well-separated clusters, consistent with their differential expression patterns and mutation-specific metabolic alterations (Figures 5B, 5E, and S5B). The comparative analysis of the typically differentially expressed metabolites revealed distinct metabolic signatures between HBV mutational groups (G1896A, A1762T/G1764A, and A1762T/G1764A + G1896A) and the WT group (Figures 5C, 5F, and S5C). Moreover, commonly dysregulated metabolites exhibited mutation-specific expression patterns, demonstrating divergent metabolic regulation between single and double mutants. The list of typically differentially expressed metabolites is provided in Table 3. These findings suggest that HBV G1896A, A1762T/G1764A, and A1762T/G1764A + G1896A infections elicit distinct metabolic regulatory mechanisms, resulting in mutation-specific metabolic profiles compared with HBV WT infections.

**Table 3:** List of typically differentially expressed metabolites between the serum samples from patients infected with HBV G1896A, A1762T/G1764A, and A1762T/G1764A + G1896A mutants

Metabolite ID	Adducted form	Metabolite name	Fold change G1896A vs. WT	Fold change A1762T/G1764A vs. WT	Fold change A1762T/G1764A + G1896A vs. WT
MEDL02208	[M+]^+^	nornicotine	0.18	0.14	0.22
ME0063042	[M-]^−^	PS(22:5(7Z,10Z,13Z,16Z,19Z)/22:5(4Z,7Z,10Z,13Z,16Z))	0.65	0.32	0.26
ME0060324	[M + HCOO] ^−^	PE-NMe2(18:0/22:4(7Z,10Z,13Z,16Z))	0.43	0.36	0.28
ME0050045	[M + H-H2O]^+^	1-stearoyl-2-docosahexaenoyl-*sn*-glycerol	0.87	0.54	0.32
ME0105425	[2M + NH4]^+^	Ac-Leu-Leu-norleucinal	0.44	0.14	0.33
MEDP0513	[M + H]^+^	palmitic amide	0.41	0.52	0.36
ME0110288	[M + H]^+^	tyrosylleucine	1.63	2.20	2.50
MEDL00638	[M-H] ^−^	taurocholic acid	0.94	2.45	2.54
ME0148244	[M + Na]^+^	delta-Carotene	3.35	5.76	3.72
ME0106005	[M + H]^+^	benazepril	3.60	2.46	3.84
ME0063621	[M-H] ^−^	sphinganine 1-phosphate	2.44	3.74	4.13
ME0053818	[M-]^−^	[(2R,3S,4S,5R,6R)-6-[[(7R,9S,10R,13R,14S,16S,17R)-3-[(2S,3R,4R,5R)-5-acetyloxy-3,4-dihydroxyoxan-2-yl]oxy-7-hydroxy-4,4,9,13,14-pentamethyl-17-[(2R)-6-methylhept-5-en-2-yl]-2,3,7,8,10,11,12,15,16,17-decahydro-1H-cyclopenta[*a*]phenanthren-16-yl]oxy]-3,4,5-trihydroxyoxan-2-yl]methyl acetate	3.40	6.51	4.25
ME0056865	[M-H] ^−^	phosphatidylcholine	1.57	4.12	4.43
ME0161662	[2M + K]^+^	(8R,10S,13S,17S)-17-hydroxy-10-(hydroxymethyl)-13-methyl-1,2,6,7,8,9,11,12,14,15,16,17-dodecahydrocyclopenta[*a*]phenanthren-3-one	2.63	5.30	5.25
MEDL02225	[M-H] ^−^	arabinose	3.77	4.01	5.45
ME0054916	[M + H]^+^	acetylacrylic Acid Methyl Ester	2.34	2.65	5.97
ME0167339	[M + Na-2H]^−^	triglyceride	1.74	4.04	6.46
MEDP0428	[2M + K]^+^	cholesterol	0.97	2.17	7.83
ME0013396	PE(18:0/20:4(5Z,8Z,11Z,14Z)	C43H78NO8P	6.47	16.32	14.32
ME0053478	[M-H] ^−^	genipostiveidic acid	58.62	19.90	24.72

### Correlation and KEGG pathway analysis of the differentially expressed metabolites

**Figure 6:**
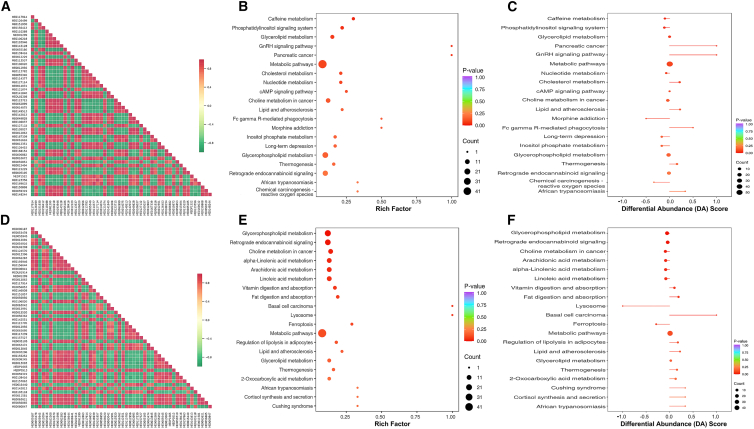
Correlation analysis of differentially expressed metabolites from patients infected with HBV A1762T/G1764A and A1762T/G1764A + G1896A mutants (A–C) Heatmap of correlation analysis (A), KEGG annotation (B), and bubble plot of KEGG pathway enrichment (C) for differentially expressed metabolites in serum samples from the HBV A1762T/G1764A mutation group and WT group (group C vs. group A). (D–F) Heatmap of correlation analysis (D), KEGG annotation (E), and bubble plot of KEGG pathway enrichment (F) for differentially expressed metabolites in serum samples from the HBV A1762T/G1764A + G1896A mutation group and WT group (group D vs. group A).

To systematically characterize serum metabolite interactions, we performed correlation analysis and clustering heatmap analysis based on Pearson’s correlation coefficients among differentially expressed metabolites. The absolute values of correlation coefficients were visualized through color intensity gradients, revealing that the majority of these metabolites exhibited positive inter-correlations, as anticipated (Figures 6A, 6D, and S5D). KEGG enrichment analysis revealed that dysregulated metabolites in the G1896A group were significantly enriched in pathways associated with D-amino acid metabolism, ferroptosis, lysosome function, selenocompound metabolism, tryptophan metabolism, and one-carbon folate metabolism (Figures S5E and S5F), whereas those in the A1762T/G1764A group were primarily linked to caffeine metabolism, phosphatidylinositol signaling, GnRH signaling pathway, pancreatic cancer, metabolic pathways, and cholesterol metabolism (Figures 6B and 6C). In contrast, the combined A1762T/G1764A + G1896A group exhibited predominant dysregulation in glycerophospholipid metabolism, retrograde endocannabinoid signaling, choline metabolism in cancer, and fatty acid metabolism pathways including alpha-linolenic acid, arachidonic acid, and linoleic acid metabolism (Figures 6E and 6F). Together, the cumulative KEGG pathway alterations observed in patients infected with HBV having distinct mutation profiles (G1896A, A1762T/G1764A, and A1762T/G1764A + G1896A) collectively suggest mutation-specific metabolic reprogramming that may contribute to the pathogenesis and disease progression of HBV-related liver disorders.

### Pathway analysis of integrating proteomic and metabolomic data of patients infected with HBV A1762T/G1764A and G1896A mutants

**Figure 7:**
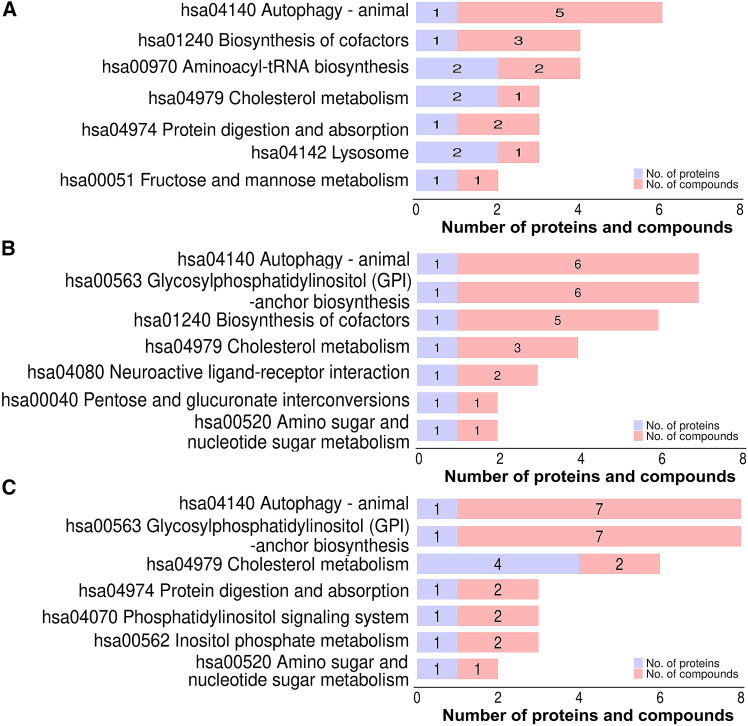
KEGG pathway analysis of integrating proteomic and metabolomic data of patient infected with HBV A1762T/G1764A and G1896A mutants (A) Mapping analysis of differentially expressed proteins and metabolites between G1896A mutation group and the WT group (group B vs. group A). (B) Mapping analysis of differentially expressed proteins and metabolites between A1762T/G1764A mutation group and the WT group (group C vs. group A). (C) Mapping analysis of differentially expressed proteins and metabolites between A1762T/G1764A + G1896A mutation group and the WT group (group D vs. group A).

**Figure 8:**
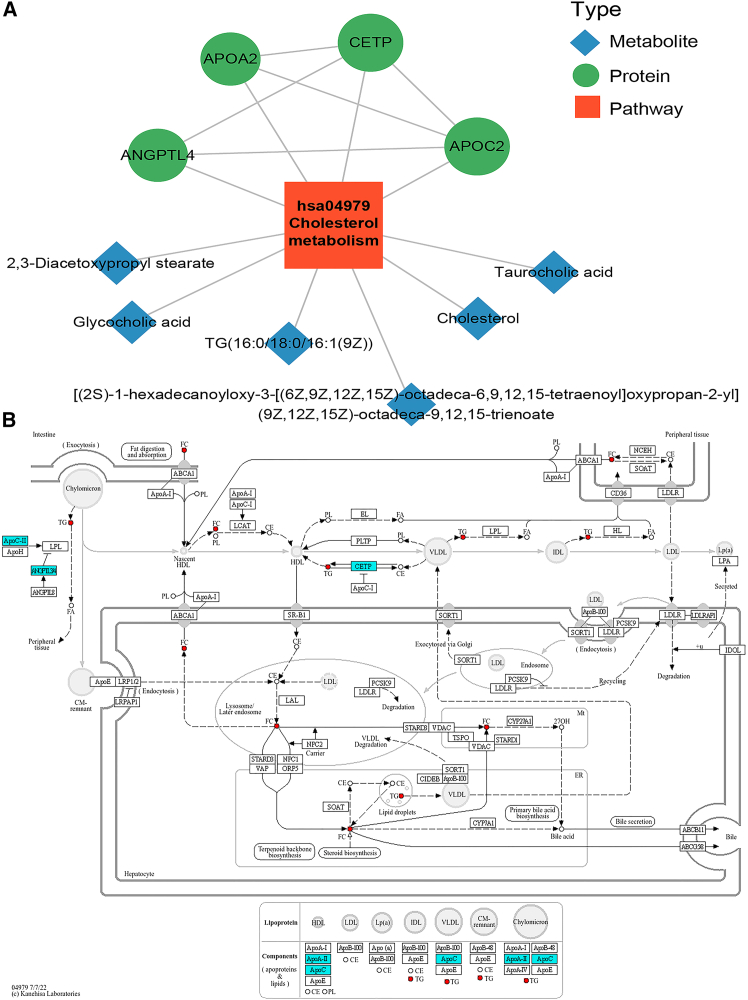
Integrated analysis of differentially expressed proteins and metabolites (A) Predictive network of differentially expressed proteins and metabolites involved in cholesterol metabolism. (B) Key signaling pathways identified through analysis of serum samples from patients infected with different HBV mutants. Cholesterol metabolism obtained from KEGG pathway-based enrichment analysis of dysregulated proteins and metabolite.

To better elucidate potential synergistic interactions between differentially expressed proteins and metabolites within biological pathways, we conducted integrated KEGG pathway enrichment analysis across both omics datasets, identifying pathways exhibiting concurrent enrichment at both the proteomic and metabolomic levels. KEGG pathway analysis revealed distinct enrichment patterns across the HBV mutant groups compared with the WT HBV group: the G1896A group showed predominant dysregulation in autophagy-animal, cofactor biosynthesis, aminoacyl-tRNA biosynthesis, cholesterol metabolism, and protein digestion/absorption (Figure 7A); the A1762T/G1764A group exhibited alterations in autophagy-animal, glycosylphosphatidylinositol (GPI)-anchor biosynthesis, cofactor biosynthesis, cholesterol metabolism, and neuroactive ligand-receptor interaction (Figure 7B); and the A1762T/G1764A + G1896A double-mutant group demonstrated significant enrichment in autophagy-animal, GPI-anchor biosynthesis, cholesterol metabolism, protein digestion/absorption, and phosphatidylinositol signaling (Figure 7C). Notably, cholesterol metabolism emerged as a consistently perturbed pathway across all mutant groups, suggesting its potential roles in the pathogenesis of HBV mutation-specific liver disease. Here, to clarify key regulatory factors in cholesterol metabolism, we constructed an interaction network integrating differentially expressed proteins and metabolites within this pathway (Figure 8A). Our analysis identified several core regulatory proteins—including angiopoietin-related protein 4 (ANGPTL4), apolipoprotein C-II (APOC2), and cholesteryl ester transfer protein (CETP)—all of which were significantly downregulated. Conversely, their associated metabolites, such as triglyceride (TG), free cholesterol (FC), and taurocholic acid (TCA), consistently showed upregulated expression (Figures 8A and 8B). Therefore, our results demonstrated that in HBV A1762T/G1764A and G1896A mutation-associated liver diseases, the cholesterol metabolism pathway may play a regulatory role in pathogenesis, disease progression, and metastasis by modulating differentially expressed proteins and metabolites.

### Verification of the selected differentially expressed proteins and metabolites

As evidenced by the key modulated signaling pathways (Figure 8) and hierarchical clustering analysis (Figures 2A, 5B, 5E, and S5B), the proteins ANGPTL4, APOC2, and CETP, along with metabolites TG, FC, and TCA, exhibited differential expression patterns between HBV mutational groups (G1896A, A1762T/G1764A, and A1762T/G1764A + G1896A) and the WT HBV group. Therefore, the differential expression of proteins (ANGPTL4, APOC2, and CETP) and metabolites (TG, FC, and TCA) may establish mutation-specific regulatory networks governing biological processes and molecular functions in patients infected with HBV with G1896A and A1762T/G1764A mutations, potentially through modulation of the cholesterol metabolism pathway. Hence, to further validate the proteomic and metabolomic findings, we quantified serum levels of the aforementioned dysregulated proteins and metabolites across four HBV cohorts: WT (*n* = 36), G1896A (*n* = 36), A1762T/G1764A (*n* = 36), and A1762T/G1764A + G1896A (*n* = 36), using multivariate analysis and enzyme-linked immunosorbent assay (ELISA) with independent sample analysis (108 total samples). Multivariate analysis adjusted for age, liver function, and viral load identified the HBV A1762T/G1764A mutation and its combination with G1896A as the strongest predictors of proteomic and metabolic changes (Tables S7 and S8). A1762T/G1764A mutation was inversely associated with the lipid-modulating proteins APOC2, ANGPTL4, and CETP but positively correlated with TG, FC, and TCA. In contrast, G1896A alone was only linked to reduced ANGPTL4. The combined mutation synergistically amplified these effects, exerting stronger downregulation of the proteins and enhancing upregulation of the metabolites. Age also contributed to variations in lipid and bile acid profiles. These cross-regulatory patterns suggested that HBV core promoter mutations are key viral determinants driving these dysregulated proteins and metabolites in HBV infection.

**Figure 9:**
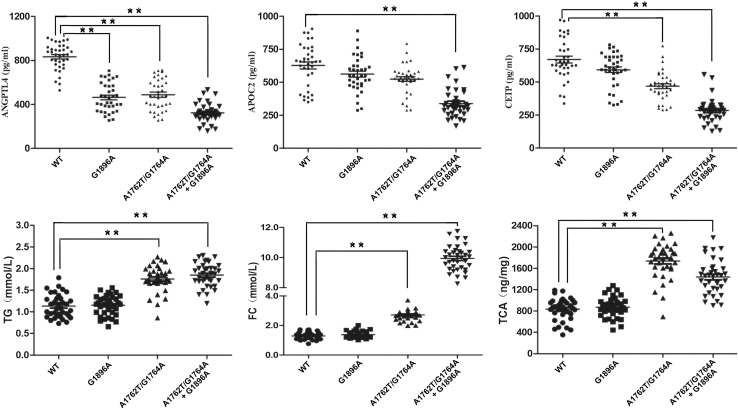
Validation of the selected differentially expressed proteins and metabolites in serum samples from patients infected with HBV A1762T/G1764A and G1896A mutants by ELISA in the validation cohort. Data are presented as the mean ± SEM; *n* = 36, ∗*p* < 0.05, ∗∗*p* < 0.01

As demonstrated in Figure 9, ELISA showed that both the A1762T/G1764A single-mutation and A1762T/G1764A + G1896A double-mutation groups exhibited significant downregulation of ANGPTL4 and CETP compared to the WT group, while the serum expression levels of APOC2 did not differ significantly between the A1762T/G1764A group and WT group. Concomitantly, serum concentrations of TG, FC, and TCA were markedly elevated in these mutant groups relative to the WT group (Figure 9). Notably, serum ANGPTL4 levels showed significant downregulation (1.79-fold change, *n* = 36, *p* < 0.01) in the G1896A group relative to the WT group, while APOC2 expression remained unchanged—findings fully consistent with our proteomic and metabolomic analysis. Thus, these results strongly suggest that these dysregulated proteins and metabolites may play pivotal roles in HBV A1762T/G1764A- and G1896A-associated liver diseases through cholesterol metabolism modulation, though the underlying molecular mechanisms require further elucidation.

**Figure 10:**
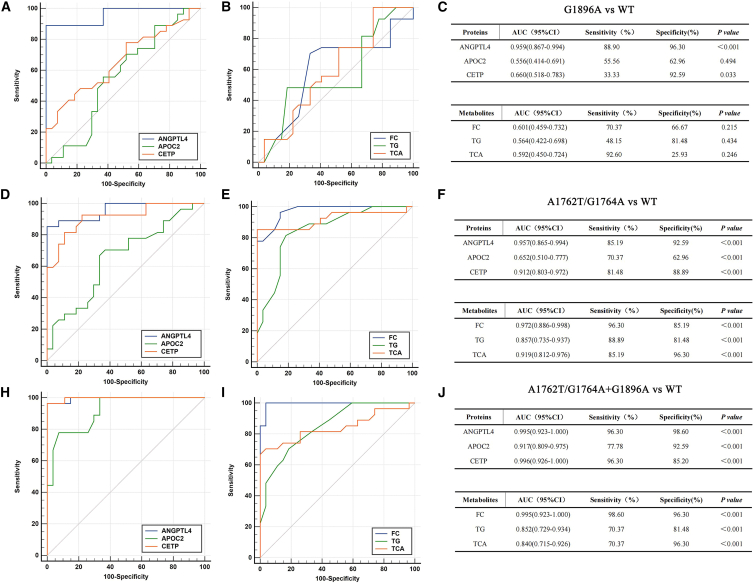
Diagnostic performance of candidate protein and metabolite biomarkers for discriminating wild-type HBV-infected patients from those infected with HBV A1762T/G1764A and G1896A mutants by ROC curve analysis in the validation cohort (A–C) ROC curve analysis of differential proteins (ANGPTL4, APOC2, and CETP) and metabolites (FC, TG, and TCA) for distinguishing between the G1896A mutant and wild-type (WT) groups, and (C) the corresponding area under the curve (AUC) values, sensitivity, and specificity. (D–F) ROC curve analysis differential proteins (ANGPTL4, APOC2, and CETP) and metabolites (FC, TG, and TCA) for distinguishing between the A1762T/G1764A mutant and WT groups, and (F) the corresponding AUC values, sensitivity, and specificity. (H–J) ROC curve analysis differential proteins (ANGPTL4, APOC2, and CETP) and metabolites (FC, TG, and TCA) for distinguishing between the A1762T/G1764A + G1896A double mutant and WT groups, and (J) the corresponding AUC values, sensitivity, and specificity.

To evaluate the diagnostic potential of the identified differential proteins (ANGPTL4, APOC2, and CETP) and metabolites (FC, TG, and TCA) in distinguishing patients infected with different HBV mutant strains from those infected with the WT strains, we performed receiver operating characteristic (ROC) curve analysis (Figure 10). The analysis revealed outstanding diagnostic performance in distinguishing the double mutant and A1762T/G1764A mutant from the WT group. Specifically, in the comparison between A1762T/G1764A + G1896A and WT, both protein and metabolite biomarkers demonstrated nearly perfect discrimination. The proteins ANGPTL4 (area under the curve [AUC] = 0.995, 95% CI: 0.923–1.000) and CETP (AUC = 0.996, 95% CI: 0.926–1.000), along with the metabolite FC (AUC = 0.995, 95% CI: 0.923–1.000), achieved exceptionally high AUC values, accompanied by sensitivity and specificity values all exceeding 96.3% and 85.2%, respectively. Likewise, when comparing A1762T/G1764A with WT, these biomarkers continued to show strong predictive power. In contrast, the ability of these differential proteins and metabolites to distinguish between the G1896A group and the WT group was inadequate, as shown by their lower AUC values and non-significant *p* values (*p* > 0.05). Ultimately, the ROC analysis robustly validates that the perturbations in cholesterol-related pathways, represented by a panel of specific proteins and metabolites, possess high diagnostic value for HBV infections involving A1762T/G1764A mutation, either alone or in combination with G1896A. However, these biomarkers show limited utility in identifying the single G1896A mutant, suggesting a distinct pathophysiological profile associated with this mutation.

## Discussion

Although global efforts to eradicate HBV have resulted in a consistent decline in the incidence of chronic infections worldwide, HBV remains a substantial public health challenge due to its severe complications, including cirrhosis and HCC.^1^^,^^2^^,^^3^^,^^34^^,^^35^ HBV DNA mutations are critical determinants of transmission efficiency, disease progression, and therapeutic outcomes, significantly impacting hepatic function and perturbing hepatocyte signaling pathways in HBV-related liver diseases.^5^^,^^6^^,^^7^^,^^8^^,^^9^ By altering viral antigenicity and modulating liver-associated host proteins and metabolites within core inflammatory pathways, HBV mutations drive the secretion of pro-inflammatory cytokines and chemokines, which promote hepatocyte death via apoptosis, pyroptosis, and ferroptosis, thereby exacerbating immune-mediated liver injury.^6^^,^^8^^,^^36^^,^^37^ HBV DNA mutations critically influence the variability of transmission efficiency, disease progression, and therapeutic response, thereby profoundly affecting hepatic function and metabolic pathways during the development of HBV-related liver diseases.^5^^,^^6^^,^^7^^,^^8^^,^^9^ Mutations in HBV DNA are not randomly distributed but are frequently clustered in specific genomic regions.^6^^,^^7^ For instance, the A1762T/G1764A and G1896A mutations are prevalent in severe liver disease cases, as these oncogenic variants synergistically promote hepatic injury progression and hepatocarcinogenesis by enhancing viral replication, evading immune responses, and disrupting metabolic pathways.^5^^,^^6^^,^^36^ Epidemiological studies have confirmed these mutations as independent perinatal transmission risk factors, strongly associated with acute-on-chronic liver failure and HCC progression compared to WT strains.^6^^,^^11^^,^^14^ Moreover, the dual mutation enhances vertical transmission via immune suppression while accelerating hepatocyte transformation through chronic inflammation and metabolic deregulation.^6^^,^^16^ Additionally, our findings, consistent with those of prior studies, demonstrate that the infection with HBV carrying A1762T/G1764A or G1896A mutation is significantly associated with adverse liver disease outcomes, as evidenced by PT prolongation and elevated TBIL levels, which suggest impaired synthetic liver function and cholestasis, respectively (Table 1). While existing research has extensively documented clinical disparities between HBV A1762T/G1764A or G1896A mutant infections and WT infections, the underlying molecular mechanisms and differential biological pathways remain poorly characterized. To our knowledge, the serum proteomic and metabolic profiles presented in this study constitute a comprehensive comparison between patients infected with HBV carrying A1762T/G1764A or G1896A mutation and WT cases at both proteomic and metabolomic levels. These findings not only corroborate prior evidence demonstrating that HBV A1762T/G1764A or G1896A mutations correlate with more severe clinical manifestations compared to WT infection but also reveal molecular distinctions between HBV mutant and WT strains. The mechanistic insights derived from this study are also poised to establish a foundational framework for elucidating mutation-specific pathology in HBV-related liver diseases.

To obtain comprehensive insights into the molecular pathogenesis of CHB with A1762T/G1764A or G1896A mutations and identify therapeutic targets, we conducted a systematic investigation of serum proteomic and metabolomic alterations, using the same biological samples. Utilizing an integrated DIA-MS and UHPLC-Q-Orbitrap MS/MS platform, we performed comprehensive proteome and metabolome profiling of serum samples across four cohorts (WT, G1896A, A1762T/G1764A, and A1762T/G1764A + G1896A) to profile dynamic changes in the global serum proteome and metabolome associated with these clinically relevant HBV variants. Among the 1,223 overlapping proteins and 1,968 (POS)/1,902 (NEG) overlapping metabolites identified, 94.66% (proteins) and 96.71% (POS)/96.21% (NEG) (metabolites) were consistently detected across all four experimental groups, demonstrating the robustness of the analytical workflow and ensuring the reliability of study conclusions (Figures 1F, 4E and 4F). Based on the identification criteria for differentially expressed proteins and metabolites, 59, 54, and 79 dysregulated proteins, along with 201, 242, and 226 dysregulated metabolites were identified in the G1896A, A1762T/G1764A, and A1762T/G1764A + G1896A groups, respectively, compared with the WT group, confirming that the pathogenesis of CHB involving these mutations is associated with distinct molecular mechanisms. Although all the signaling pathways associated with the abovementioned differentially expressed proteins and metabolites are key players in the course of infection with HBV A1762T/G1764A and G1896A mutants, the cholesterol metabolism pathway emerged as a consistently dysregulated pathway across all experimental groups, with enrichment of key dysregulated proteins and metabolites at the core of the PPI network (Figure 7). This finding suggests that in CHB patients infected with HBV harboring A1762T/G1764A and G1896A mutations, the cholesterol metabolism pathway may play a critical role in modulating clinical features and disease progression during HBV infection, potentially serving as a therapeutic target for CHB treatment.

Cholesterol, an essential sterol for mammalian cell proliferation, exhibits high dynamism and interacts with adjacent lipids to modulate membrane rigidity, fluidity, and permeability, playing multifaceted roles in cellular homeostasis, viral entry, replication, and assembly.^38^^,^^39^^,^^40^ As a crucial precursor of various steroid hormones, cholesterol homeostasis disruption has been extensively documented to impair normal cellular and systemic physiology, with established associations with cardiovascular diseases, neurodegenerative disorders, autoimmune conditions, and various malignancies.^38^^,^^40^ When intracellular cholesterol levels decline, the sterol-sensing pathway triggers the proteolytic activation of sterol regulatory element-binding protein isoform 2 (SREBP-2), a master transcriptional regulator that subsequently translocates to the nucleus and binds to sterol response elements (SREs) in the promoters of genes encoding two key mevalonate pathway enzymes: 3-hydroxy-3-methylglutaryl-CoA reductase (HMGCR) and squalene epoxidase (SQLE).^41^^,^^42^ Conversely, when cellular cholesterol concentrations exceed physiological thresholds, cholesterol accumulation triggers ubiquitin-mediated degradation of both HMGCR and SQLE, while simultaneously inhibiting SREBP-2 processing, thereby completing the feedback loop that maintains cholesterol homeostasis.^41^^,^^42^^,^^43^ Accordingly, dysregulated cholesterol metabolism, a tightly regulated process essential for membrane integrity and hormone synthesis, serves as a pathogenic driver in HBV infection by disrupting lipid raft signaling critical for cellular transformation.^40^^,^^43^ In this context, it transcends its conventional homeostatic role to critically govern viral dissemination and disease progression.^44^ *In vitro* studies have delineated a dual-pronged mechanism whereby HBeAg directly perturbs cholesterol homeostasis: it activates biosynthesis by disrupting ER retention of the SCAP-SREBP complex while concurrently suppressing LXR-mediated efflux and catabolism, resulting in net intracellular accumulation.^44^^,^^45^ HBeAg may further engage FXR and peroxisome proliferator-activated receptors (PPARs) to orchestrate broader lipid metabolic reprogramming.^42^^,^^45^ Additionally, HBeAg indirectly modulates cholesterol metabolism by activating NF-κB and JAK/STAT pathways to stimulate cytokine-driven, paracrine regulation of SREBPs and LXRs, with precore mutations potentially introducing functional heterogeneity in these nuclear receptor interactions that contributes to the metabolic diversity observed in CHB.^42^^,^^45^ Therefore, our data support that the clinical outcomes of infections with HBV carrying A1762T/G1764A and G1896A mutations are critically influenced by host systemic cholesterol metabolism homeostasis, demonstrating that the cholesterol metabolic signaling pathway is directly engaged following infection with these HBV variants.

Furthermore, our analysis revealed that the proteins ANGPTL4, APOC2, and CETP were significantly downregulated in the serum samples from CHB patients with A1762T/G1764A or G1896A mutants compared to those carrying WT HBV strains, which reflects potentially distinct pathophysiological mechanisms associated with these viral mutations. For example, ANGPTL4, a member of the eight-protein angiopoietin-like family (ANGPTLs) that shares structural homology with angiopoietins (ANGPTs), acts as a key metabolic regulator by potently inhibiting lipoprotein lipase (LPL) activity.^46^ This inhibition blocks TG hydrolysis in lipoproteins, while simultaneously exhibiting an inverse correlation with high-density lipoprotein cholesterol (HDL-C), thereby collectively influencing lipid distribution and cholesterol homeostasis.^47^ In the context of liver fibrosis, ANGPTL4 regulates cholesterol accumulation in hepatic stellate cells (HSCs) by inhibiting LPL, and its deficiency exacerbates fibrosis by sensitizing HSCs to TGF-β-induced activation independent of hepatocellular injury or inflammation.^48^ Notably, despite its mechanistic role in TG metabolism, serum ANGPTL4 concentrations showed no significant positive association with plasma TG levels in some studies.^46^^,^^47^ Additionally, APOC2, a protein associated with both high-density lipoproteins (HDLs) and very low-density lipoproteins (VLDLs), functions as a cofactor for LPL by stimulating the hydrolysis of TGs into fatty acids within these lipoproteins.^49^ Beyond its canonical role in lipid metabolism, APOC2 modulates HSC activation through a dual regulatory pathway: it influences peroxisome proliferator-activated receptor gamma (PPARγ), a nuclear receptor critical for maintaining HSC quiescence and suppressing fibrogenesis, and concurrently regulates the transforming growth factor β (TGF-β)/Smad signaling cascade, thereby governing HSC phenotypic transformation.^49^^,^^50^ However, APOC2 exhibits concentration-dependent regulation of LPL activity—activating it at low concentrations while paradoxically inhibiting it at high levels through a mechanism likely requiring LPL binding to phospholipids on TG-rich lipoprotein surfaces as a prerequisite for APOC2-mediated activation, though this process remains incompletely understood.^51^^,^^52^ By competing with ANGPTL4 for LPL binding, APOC2 prevents LPL inactivation, indirectly enabling CETP to promote cholesterol ester (CE)-to-TG conversion through accelerated TG breakdown and thus lowering blood lipids; CETP, a hepatic plasma glycoprotein released into circulation, critically modulates reverse cholesterol transport and plasma lipid distribution.^53^^,^^54^ Consistent with these existing literatures, our observations indicated that the downregulation of ANGPTL4, APOC2, CETP and other soluble regulators of cholesterol metabolism involved in LPL activity and cholesterol homeostasis, may contribute to enhanced inactivation of cholesterol metabolic signaling, with this effect being significantly more pronounced in serum samples from HBV-infected patients carrying A1762T/G1764A and G1896A mutations compared to WT HBV strains. Therefore, the coordinated suppression of ANGPTL4 that inhibits LPL-mediated HDL catabolism, APOC2 that concentration-dependently regulates LPL activity, and CETP that disrupts CE transfer suggests that HBV A1762T/G1764A and G1896A mutations dysregulate cholesterol metabolic signaling, thereby significantly increasing the risk of more aggressive HBV-related liver disease.

In untargeted metabolomics data, we noted a marked increase in FC, TG, and TCA levels in HBV serum from the A1762T/G1764A and A1762T/G1764A + G1896A mutant groups compared with the WT group, suggesting a significant disturbance in cholesterol balance. The pronounced elevation in FC is of particular significance, given the growing recognition that excess FC acts as an active contributor to metabolic and inflammatory diseases, rather than merely a passive biomarker of cellular dysfunction and pathogenesis.^55^^,^^56^ Excessive FC can be incorporated into cell membranes, impacting their fluidity and signaling abilities and leading to ER stress, inflammation, and apoptosis, which are key factors for the progression of atherosclerosis.^55^ The concomitant elevation in TG points to dysregulated lipid storage and mobilization, potentially stemming from enhanced synthesis or impaired lipolysis, thereby exacerbating cellular lipid overload and lipotoxicity.^57^^,^^58^ Concurrently, the marked rise in the primary bile acid TCA indicates a shift in bile acid metabolism, a pathway critical for cholesterol disposal.^59^^,^^60^ This TCA upregulation likely represents an adaptive hepatic response to elevated cholesterol, aimed at promoting its excretion, yet sustained high levels of bile acids can induce hepatocyte oxidative stress and inflammation, indicating that initial compensatory mechanisms may transition toward maladaptation.^38^^,^^55^^,^^59^ The concurrent elevation of TG, FC, and TCA suggests a coordinated yet dysregulated metabolic response, potentially driven by enhanced cholesterol biosynthesis, impaired reverse cholesterol transport, or disrupted hepatic lipid metabolites.^38^^,^^57^ The present study identified dysregulation of several proteins and metabolites, including ANGPTL4, APOC2, CETP, FC, TG, and TCA, which are functionally associated with the cholesterol metabolism pathways. These pathways may play a regulatory role in HBV-related liver diseases by modulating serum levels of these proteins and metabolites. Consequently, the expression levels of these differentially regulated proteins and metabolites could serve as potential biomarkers for distinguishing between patients infected with the HBV variants A1762T/G1764A or A1762T/G1764A + G1896A and the WT HBV. Moreover, these serum protein and metabolite markers may also represent potential therapeutic targets for infections caused by HBV A1762T/G1764A and A1762T/G1764A + G1896A mutants, thereby aiding in the prevention and control of HBV infections and associated liver diseases. However, the underlying mechanisms by which these proteins and metabolites regulate cholesterol metabolism pathways remain to be fully elucidated, warranting further investigation.

Overall, our study employed an integrated multi-omics approach, combining DIA proteomics with untargeted metabolomics, to analyze serum samples from patients infected with HBV A1762T/G1764A and A1762T/G1764A + G1896A mutants, alongside WT HBV-infected controls. This comprehensive analysis successfully identified distinct protein and metabolite signatures specifically associated with CHB patients, characterized their expression profiles and co-regulatory networks, and revealed that dysregulated cholesterol metabolism plays a pivotal role in the pathogenesis of HBV infections. As anticipated, the results demonstrated distinct serum proteomic and metabolic fingerprints in individuals infected with different HBV variants, indicating that the A1762T/G1764A and A1762T/G1764A + G1896A mutant strains differentially reprogram host cellular metabolism. Using the same omics platforms, we confirmed consistent detection of three proteins (ANGPTL4, APOC2, and CETP) and three metabolites (FC, TG, and TCA) across replicates, all associated with the cholesterol metabolism pathway, thereby demonstrating methodological reliability. This integrated proteomic and metabolomic investigation delineates the physiological impact of HBV variants (A1762T/G1764A and A1762T/G1764A + G1896A), providing mechanistic insights that significantly advance our understanding of HBV-related pathogenesis, and our results underscore the cholesterol metabolic pathway as a promising therapeutic target for diseases driven by these HBV variants, potentially amenable to intervention through modulation of key metabolic biomarkers.

### Limitations of the study

Analyzing alterations in the serum proteome and metabolome, rather than in liver tissues, might have limited our ability to fully capture tissue-specific cellular phenomena. Second, the cross-sectional design and relatively limited sample size may affect the generalizability of the finding. Third, although multi-omics profiling provides a broad view, the current data are primarily descriptive; the absence of functional validation and assessment for potential confounders—such as viral genotype, age, sex or gender, mutation abundance, and tissue-specific viral heterogeneity—restricts mechanistic interpretation. Fourth, the high discriminative performance of certain biomarkers in this cohort may reflect overfitting, necessitating validation in larger, independent populations. Finally, despite stringent controls, inherent technical variability in omics methodologies could introduce residual confounding. Future longitudinal studies incorporating functional assays and multicenter cohorts are needed to confirm these findings and establish causality.

## Resource availability

### Lead contact

Requests for further additional information and resources should be directed to and will be fulfilled by the lead contact, Dahai Wei (weidahai3166@hhotmail.com).

### Materials availability

The study did not generate new unique reagents and materials.

### Data and code availability

#### Data

The mass spectrometry proteomics data generated in this study have been deposited to the ProteomeXchange Consortium via the PRIDE partner repository, with the dataset identifier: PXD066939. The dataset is publicly accessible at: https://www.ebi.ac.uk/pride/archive/projects/PXD066939. Untargeted metabolomics data are available within the article and its supplemental information files. Any additional data required to reanalyze the findings reported in this paper are available from the lead contact upon reasonable request.

#### Code

This study does not report original code.

#### Additional information

Any additional information required for reanalyzing the data reported in this paper is available from the lead contact upon request.

## Acknowledgments

This work was supported by 10.13039/501100004731Zhejiang Provincial Natural Science Foundation of China (under grant no. LTGY24C010001), Jiaxing Key Laboratory of Virus-related Infectious Disease, and 2023 Jiaxing Key Discipline of Medicine-Lemology (Supporting Subject, grant no. 2023-ZC-009).

## Author contributions

Conceptualization, D.W. and M.D.; methodology, Y.C. and D.W.; investigation, Y.C., P.L, J.J., and D.W.; writing – original draft, Y.C. and D.W.; writing – review & editing, D.W. and M.D.; funding acquisition and resources, M.D. and D.W.; supervision, D.W. and M.D. All authors have reviewed and approved the final manuscript.

## Declaration of interests

The authors declare no competing interests.

## Declaration of generative AI and AI-assisted technologies in the writing process

The authors did not use generative AI or AI-assisted technologies in the preparation of this work.

## STAR★Methods

### Key resources table

**Table:** 

REAGENT or RESOURCE	SOURCE	IDENTIFIER
**Chemicals, peptides, and recombinant proteins**		

DTT	Sigma	Cat#43815
TEAB	Sigma	Cat#19297
Trypsin	Promega	Cat#V5280
Dimethylbenzene	Sangon Biotech	Cat#A501469
TCA	Sigma	Cat#T6399
Protease inhibitor cocktail V	Merck Millipore	Cat#535140
Waters ACQUITY Premier HSS T3 Column (2.1 mm × 100 mm, 1.8 μm)	Waters	Cat#186009452

**Biological samples**		

Serum samples	This study	N/A

**Critical commercial assays**		

Pierce™ Top 14 Abundant Protein Depletion Spin Columns Kit	ThermoFisher	Cat#A36470
BCA assay kit	Bio-Rad	Cat#500112
EASY-nLC 1200	ThermoFisher	Cat#LC140
Pierce High pH Reversed-Phase Peptide Fractionation Kit	ThermoFisher	Cat#84868

**Deposited data**		

Proteomics raw data (DIA LC-MS/MS)	This study	ProteomeXchange Consortium via PRIDE: PXD066939 Publicly accessible at: https://www.ebi.ac.uk/pride/archive/projects/PXD066939

**Software and algorithms**		

Spectronaut software	Biognosys AG	SpectronautTM 18.2.240610.53300
ProteinPilot software	AB SCIEX	Version 5.0
SnapGene Viewer	Insightful Science	Version 6.02
MedCalc	MedCalc Software	Version 22.0
SPSS Statistics	IBM	Version 23.0
ProteoWizard MSConvert software	N/A	http://proteowizard.sourceforge.net/
XCMS Online	N/A	https://xcmsonline.scripps.edu
Full GO term annotation	Gene Ontology Consortium	http://current.geneontology.org/products/pages/downloads.html
Blast2GO bioinformatics platform	BioBam Bioinformatics	http://www.blast2go.com/b2ghome
KEGG database	N/A	http://geneontology.org/
MetaboAnalyst 6.0	N/A	https://dev.metaboanalyst.ca/MetaboAnalyst/upload/JointUploadView.xhtml
GraphPadPRISM8.0	GraphPadSoftware	https://www.graphpad.com/ scientific-software/prism/

### Experimental model and study participant details

The experimental model in this study was human participants. All participants were of Chinese ancestry/ethnicity. A total of 252 treatment-naive patients with chronic HBV infection were enrolled. Participants were stratified into four groups based on HBV mutation status: wild-type (WT, *n* = 27), G1896A mutant (*n* = 27), A1762T/G1764A mutant (*n* = 27), and A1762T/G1764A + G1896A double mutant (*n* = 27). The demographic and clinical characteristics of all participants are detailed in Table 1.

• Species/Strain: Homo sapiens (Chinese population).
• Genotype: Not applicable.
• Age/Developmental Stage: Adults aged 18 to 65 years.
• Sex: The sex distribution (male/female) for each group is provided in Table 1.
• Gender, Ancestry, Race, and Ethnicity: Gender identity was not separately reported. All participants were of Chinese ancestry, which encompasses their race and ethnicity.
• Maintenance/Care: Not applicable.
• Institutional Permission and Oversight: This study was conducted in accordance with the Declaration of Helsinki. Written informed consent was obtained from all participants. The study protocol was reviewed and approved by the Ethics Committee of the Affiliated Hospital of Jiaxing University (Approval Number: 2024-LY-727).

### Method details

#### Patients and sample collection

The study sample consisted of CHB patients who visited the Department of Infectious Diseases at Affiliated Hospital of Jiaxing University between January 1 and December 31, 2024. All individuals, aged 18 to 65, satisfied the diagnostic criteria for chronic HBV infection as outlined in the 2022 Chinese Guidelines for CHB Prevention and Treatment. Analysis of A1762T/G1764A and G1896A mutations was characterized by the Sanger sequencing method using gene-specific primers (forward, 5′-GTGTGCACTTCGCTTCACC-3′ and reverse, 5′-ATAGCTTGCCTGAGTGCCGTAT-3’). Mutation analysis was carried out by alignment against the standard sequences of HBV NCBI reference sequence (LC882187.1) using SnapGene Viewer (v6.0.2). All reported mutations were confirmed by bidirectional sequencing. Patients were excluded if, within six months prior to enrollment, they had received antiviral therapy for HBV, had confirmed co-infections with other viruses, (e.g., HAV, HCV), presented with significant comorbidities (including malignancies such as HCC, severe fatty liver disease, diabetes mellitus, autoimmune disorders, or thyroid dysfunction), were pregnant, or had active neuropsychiatric conditions. The anonymized baseline clinical and biochemical characteristics of patients are detailed in Table 1, while the workflow chart of this study is presented in Figure S1.

Ultimately, a total of 252 CHB patients (108 test cohort and 144 validation cohort) were enrolled and categorized into four groups according to their sequencing results: patients infected HBV WT (Group A, *n* = 27), patients infected HBV G1896A mutation (Group B, *n* = 27), patients infected with HBV A1762T/G1764A mutation (Group C, *n* = 27), and patients infected with HBV A1762T/G1764A + G1896A mutations (Group D, *n* = 27). To minimize preanalytical variation, serum samples were systematically collected and processed according to strict standard operating procedures, aliquoted, and stored at −80°C. For each group, every 9 individual serum samples of equal volume were pooled to mitigate patient-to-patient variation for the subsequent experimental procedures.

#### DIA LC-MS/MS-based serum proteomic analysis

##### Protein extraction and trypsin digestion

Serum samples were centrifuged at 12,000 × g for 10 min at 4°C to remove cell debris, after which the supernatant was transferred to a new tube and subjected to high-abundance protein depletion using the Pierce Top 14 Abundant Protein Depletion Spin Columns Kit (ThermoFisher Scientific, Massachusetts, USA) according to the manufacturer’s instructions, prior to determining the protein concentration using a BCA assay kit (Bio-Rad, USA). For digestion, proteins were reduced using 5 mM dithiothreitol (DTT) for 30 min at 56°C, followed by alkylation with 11 mM iodoacetamide (IAA) under room temperature for 15 min in the dark. The mixture was further diluted with 100 mM triethylammonium bicarbonate (TEAB) to reduce urea concentration below 2 M, followed by tryptic digestion in two steps: first at 1:50 enzyme-to-protein ratio overnight, then at 1:100 ratio for 4 h. Finally, the resulting peptides were desalted using a C18 solid-phase extraction (SPE) column (Sigma, USA), and subsequently dried under vacuum.

##### DIA-based LC-MS/MS

After trypsin digestion, the resulting peptides were fractionated in solvent A (0.1% formic acid, 2% acetonitrile in water) and separated on a homemade reversed-phase analytical column (25 cm length,100 μm ID) using an EASY-nLC 1200 UPLC system (ThermoFisher Scientific, Massachusetts, USA), operating in DIA modes. Separation was performed with a mobile phase of solvent A and solvent B (0.1% formic acid, 90% acetonitrile in water) at a constant flow rate of 500 nL/min, using the following gradient program: 6–20% B over 0–16 min, 20–32% B over 16–24 min, 32–80% B over 24–27 min, and 80% B over 27–30 min. The eluted peptides were ionized via a nano-electrospray source operated at 2.1 kV and analyzed using an Orbitrap Exploris 480 mass spectrometer (Thermo Fisher Scientific, Massachusetts, USA). For full MS scans, spectra were acquired over the m/z range of 350–1050 at a resolution of 30,000, while MS/MS scans were performed at 450,000 resolutions with a fixed first mass at 200.0 m/z, employing higher-energy collisional dissociation (HCD) fragmentation at normalized collision energies (NCE) of 25%, 30%, and 35%. The automatic gain control (AGC) target was set to 3E6, with a maximum injection time set to Auto.

##### Proteomic data processing and analysis

The raw DIA data were further processed and analyzed using Spectronaut software (version 18; Biognosys AG, Switzerland), with tandem mass spectra searched against a combined database of Homo_sapiens_9606_SP_20241202.fasta (20,422 entries) and its reverse decoy counterpart. For the key software parameters, Trypsin/P digestion (max 2 missed cleavages) was employed with fixed carbamidomethylation on cysteine, while N-terminal acetylation and methionine oxidation were set as variable modifications, maintaining false discovery rate (FDR) < 1% across protein, peptide, and PSM levels. Proteins were considered to be significantly differentially expressed if they exhibited a fold change ≥1.5 (upregulated) or ≤0.67 (downregulated), with a statistically significant paired *t* test *p*-value <0.05.

#### UHPLC-Q-orbitrap MS/MS-based serum metabolomics analysis

##### Extraction of compounds

The metabolite extraction procedure was conducted as per the previously described methodology.^10^ In brief, the sample was prepared by mixing 50 μL of sample with 300 μL extraction solution (acetonitrile/methanol 1:4, v/v) containing internal standards. After vortexing for 3 min, followed by centrifugation at 14,000 × g for10 min at 4°C, the supernatant underwent cryoprecipitation at −20°C for 30 min. The sample was then re-centrifuged 14,000 × g for 3 min at 4°C, and 180 μL of resulting solution was injected into the LC-MS system for UHPLC-Q-Orbitrap MS/MS analysis.

##### UHPLC-Q-orbitrap MS/MS analysis

UHPLC-Q-Orbitrap MS/MS analysis was conducted using Vanquish UHPLC system (Thermo Fisher Scientific, Massachusetts, USA) equipped with Waters ACQUITY Premier HSS T3 Column (2.1 × 100 mm, 1.8 μm) connected to a Q-Exactive HF-X mass spectrometer (Thermo Fisher Scientific, Massachusetts, USA) using a linear gradient of 0.1% formic acid in water (solvent A) and 0.1% formic acid in acetonitrile (solvent B) for both positive (POS) and negative (NEG) ionization modes. The elution gradient was initiated at 5% solvent B, linearly increased to 20% solvent B in 2 min, then ramped to 60% solvent B over 3 min, further elevated to 99% solvent B in 1 min and held for 1.5 min, finally reduced to 5% solvent B in 0.1 min with 2.4 min re-equilibration, using 4 μL injection volume at 0.4 mL/min flow rate.

The analytical methods employed alternating full-scan MS and data-dependent MSn scans with dynamic exclusion enabled. Electrospray ionization (ESI) was utilized in both POS and NEG ionization modes, with full-scan mass spectra acquired across the m/z range of 75–1000 at a resolution of 35,000.Additional MS settings of ESI source conditions were as follows: ion spray voltage floating, 3.5 KV (POS) or 3.2 KV (NEG); aux gas, 5 Arb; sheath gas,30 Arb; vaporizer temperature, 300°C; ion transfer tube temperature, 320°C; collision energy, 30-40-50 V; top N vs. top speed, 10; and exclusion duration, 3s.

#### Metabolomics data processing and analysis

Raw MS data acquired from UHPLC-Q-Orbitrap MS/MS were converted to mzXML format using ProteoWizard MSConvert software, then processed with XCMS Online (https://xcmsonline.scripps.edu) for peak picking, alignment, and retention time correction using default parameters. The acquired datasets were subjected to multivariate statistical analysis of PCA, partial least squares discriminant analysis (PLS-DA), and orthogonal partial least-squares discrimination analysis (OPLS-DA) for differential metabolite screening, with model performance evaluated using R^2^ (goodness of fit) and Q^2^ (cross-validated predictive capability). Based on OPLS-DA discrimination model, the VIP score was employed as a key metric to quantify the relative contribution of each variable to the classification mode. For two-group analysis, differential metabolites were determined by VIP (VIP value >1) with a statistically significant paired *t* test *p*-value <0.05.

#### Functional analysis of the differentially expressed proteins and metabolites

Differentially expressed proteins and metabolites, which screened on statistical significance and fold-change thresholds, were subsequently visualized through hierarchical clustering using the “heatmap” function. For the classified proteins and metabolites, the Blast2GO bioinformatics platform (http://www.blast2go.com/b2ghome) was employed to conduct GO term classification, systematically analyzing protein functions across three ontological categories: BP, CC, MF. Protein and metabolite families were functionally annotated using the KEGG database (http://geneontology.org/), with subsequent enrichment analysis mapped to KEGG Pathway entries (http://www.kegg.jp/kegg/pathway.html).

#### Correlation analysis of proteomics and metabolomics

To further systematically integrate the proteomic and metabolomic datasets, we utilized the MetaboAnalyst 6.0’s pathway-level module (https://dev.metaboanalyst.ca/MetaboAnalyst/upload/JointUploadView.xhtml) to examine the relationships between differentially expressed proteins and metabolites. The integrated analytical workflow comprised two-way orthogonal partial least squares (O2PLS) regression for cross-omics dimension reduction, functional annotation enrichment analysis (KEGG/GO pathways), Venn-based intersection quantification of differentially expressed entities, and expression-metabolite correlation network construction.

#### Verification of the selected differential expression of proteins and metabolites

To validate the proteomic and metabolic findings, key differentially expressed serum proteins and metabolites were quantitatively assessed using ELISA and automated biochemical analysis, following standardized protocols as previously described.^29^^,^^33^ For the selected dysregulated proteins and metabolites, serum samples from all four groups were analyzed using a commercial ELISA kit targeting the identified dysregulated proteins and metabolites, with strict adherence to the manufacturer’s instructions. Each sample was tested in triplicate, with optical density measured at 450 nm using a BioTek ELx800 microplate reader. Protein and metabolite concentrations were automatically derived from standard curves with consideration of dilution factors, demonstrating high reproducibility (inter- and intra-assay CV < 5%). To further validate the identified proteins and metabolites, ROC curves analysis was performed using MedCalc software to assess their diagnostic utility. This included calculation of AUC, along with sensitivity and specificity metrics.

### Quantification and statistical analysis

Quantitative data (mean ± SD) were derived from ≥3 independent replicates. Group comparisons were performed using Student’s *t* test, and Spearman’s rank correlation test evaluated variable associations. Statistical significance was defined as a two-tailed *p* < 0.05. Analysis was performed with SPSS Statistics software version 23.0 (IBM Corporation, Armonk, NY, USA) for statistics and GraphPad Prism software version 9.0 for data visualization.

## Supplemental information

Document S1. Figures S1–S5

Table S1. Differentially expressed proteins of serum samples between the HBV G1896A mutation group and the WT group

Table S2. Differentially expressed proteins of serum samples between the HBV A1762T/G1764A mutation group and the WT group

Table S3. Differentially expressed proteins of serum samples between the HBV A1762T/G1764A + G1896A mutation group and the WT group

Table S4. Differentially expressed metabolites of serum samples between the HBV G1896A mutation group and the WT group

Table S5. Differentially expressed metabolites of serum samples between the HBV A1762T/G1764A mutation group and the WT group

Table S6. Differentially expressed metabolites of serum samples between the HBV A1762T/G1764A + G1896A mutation group and the WT group

Table S7. Factors that independently influence the selected differentially expressed proteins were discovered through multiple linear regression analysis

Table S8. Factors that independently influence the selected differentially expressed metabolites were discovered through multiple linear regression analysis
