# Supplementary material for: Cholesterol metabolism dysregulated by key HBV mutations revealed through multi-omics profiling
Source: iScience. 2026 Apr 22;29(5):115844. doi: 10.1016/j.isci.2026.115844 (PMC13157172; doi:10.1016/j.isci.2026.115844)
Supplement: Document S1. Figures S1–S5 [file mmc1.pdf]

iScience, Volume *29*

Supplemental information

Cholesterol metabolism dysregulated by key HBV mutations revealed through multi-omics profiling

Yimin Chen, Peixia Lin, Jiaxin Jin, Min Deng, and Dahai Wei

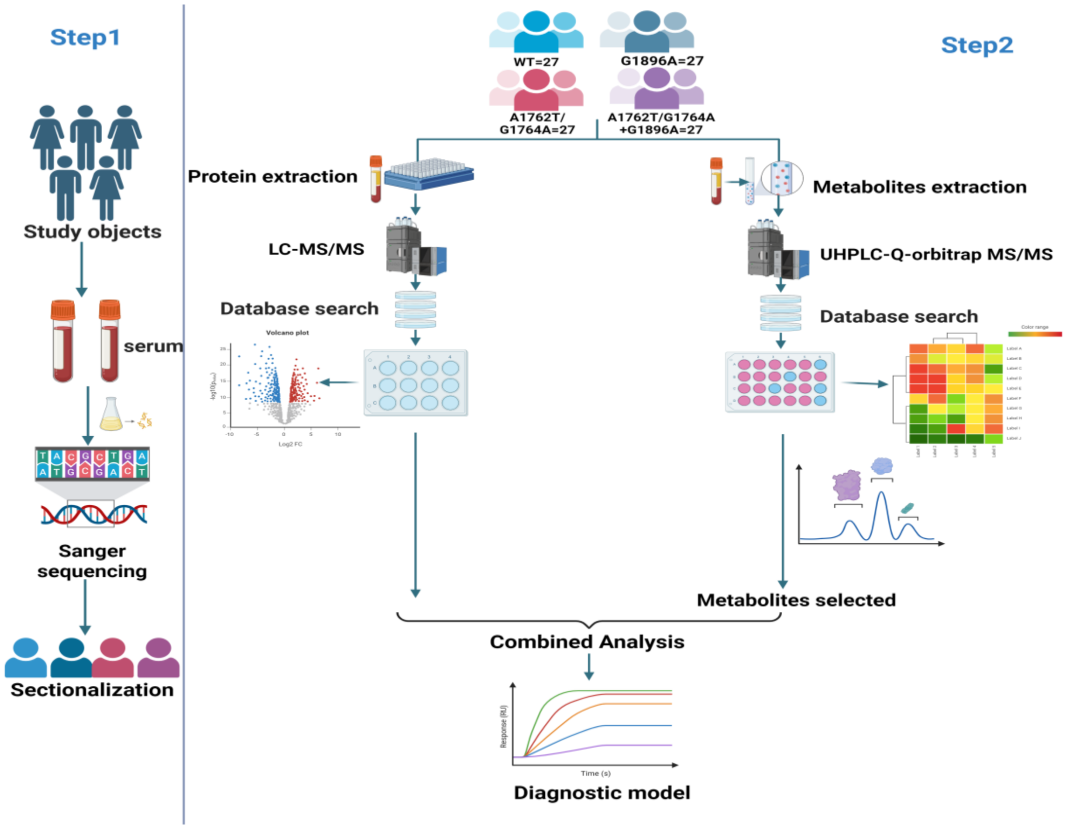

Figure S1. The overall workflow route of this project.

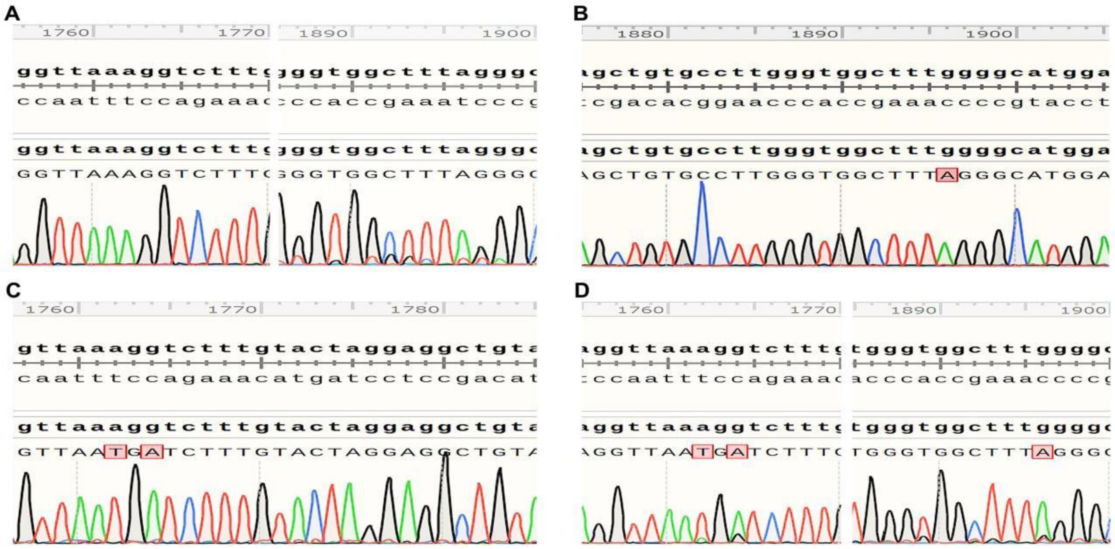

Figure S2. Characterization of HBV BCP/PC region mutations by sequence chromatogram. (A) Wild-type (WT): No characteristic mutations. (B) G1896A: A single G-to-A substitution at nucleotide 1896. (C) A1762T/G1764A: Dual substitutions of A-to-T at nt1762 and G-to-A at nt1764. (D) A1762T/G1764A + G1896A: Combined triple substitutions at nt1762 (A→T), nt1764 (G→A), and nt1896 (G→A).

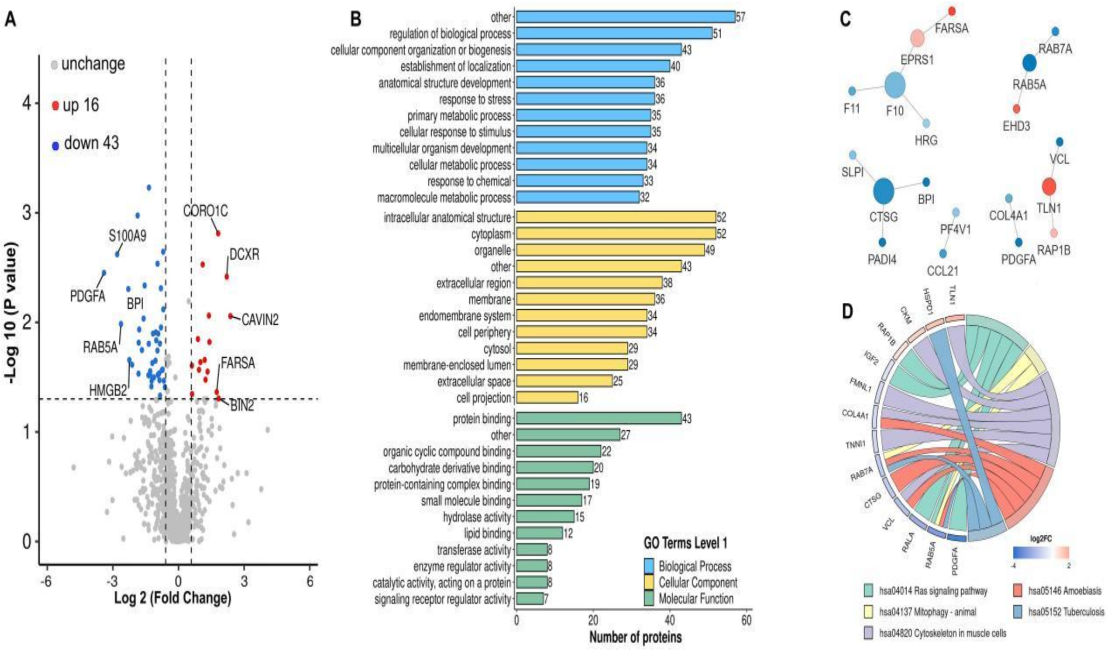

Figure S3. Bioinformatic analysis of differentially expressed proteins in the HBV G1896A mutation group. (A) Volcano plot of differentially abundant proteins in serum between patients infected with HBV G1896A mutant (Group B) and wild-type controls (Group A).(B) Gene Ontology (GO) enrichment analysis for biological processes of the differentially abundant proteins. (C) Protein-protein interaction (PPI) network of the differentially abundant proteins. (D) KEGG pathway enrichment analysis mapped onto the PPI network.

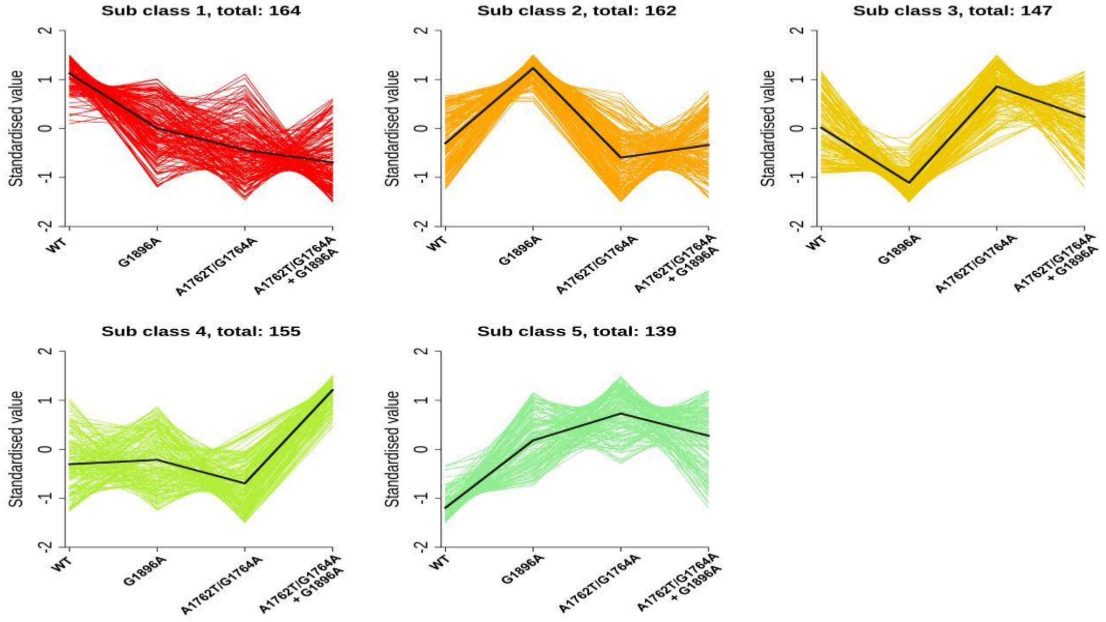

Figure S4. Cluster analysis of metabolite relative abundance trends across experimental groups using K-Means.

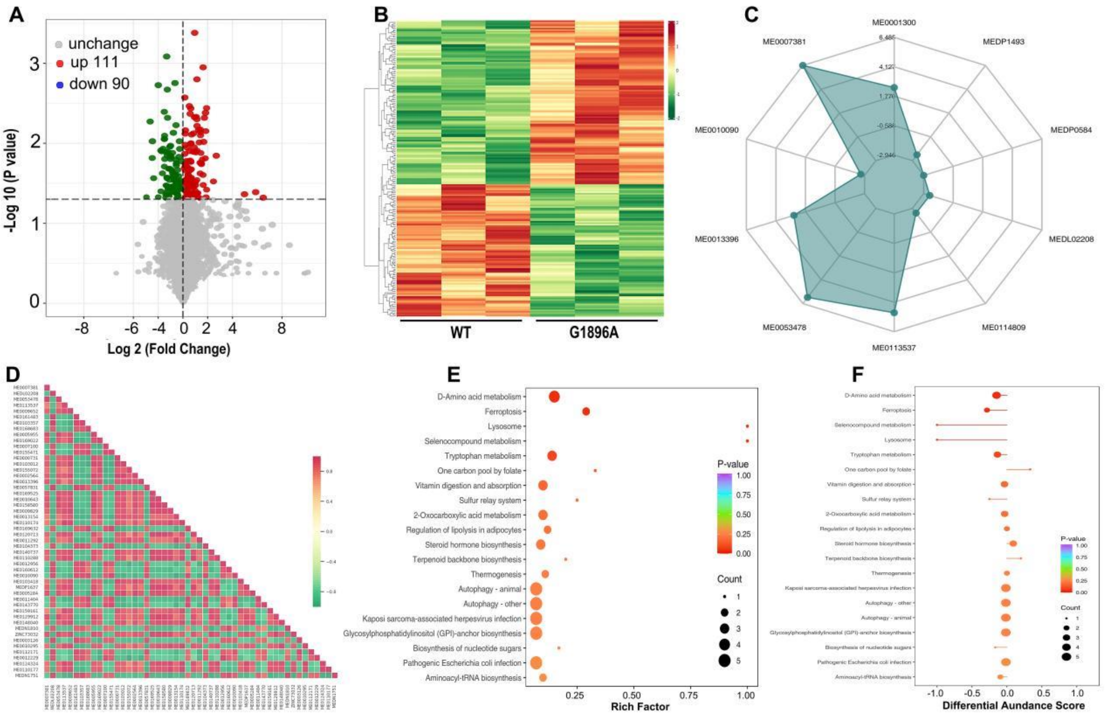

Figure S5. Bioinformatic analysis of differentially expressed metabolites in the HBV G1896A mutation group. (A) Volcano plot of differential metabolites. (B) Heatmap of hierarchical clustering analysis for differential metabolites. (C) Radar chart displaying the top 10 significantly altered metabolites. (D) Correlation heatmap of the top 50 differential metabolites ranked by VIP scores. (E) Bubble plot of KEGG pathway enrichment analysis for differential metabolites. (F) Differential Abundance Score (DAS) plot of perturbed metabolic pathways.
